# A conceptual framework for implementation fidelity

**DOI:** 10.1186/1748-5908-2-40

**Published:** 2007-11-30

**Authors:** Christopher Carroll, Malcolm Patterson, Stephen Wood, Andrew Booth, Jo Rick, Shashi Balain

**Affiliations:** 1 School of Health and Related Research (ScHARR), University of Sheffield, Sheffield, UK; 2 Institute of Work Psychology, University of Sheffield, Sheffield, UK

## Abstract

**Background:**

Implementation fidelity refers to the degree to which an intervention or programme is delivered as intended. Only by understanding and measuring whether an intervention has been implemented with fidelity can researchers and practitioners gain a better understanding of how and why an intervention works, and the extent to which outcomes can be improved.

**Discussion:**

The authors undertook a critical review of existing conceptualisations of implementation fidelity and developed a new conceptual framework for understanding and measuring the process. The resulting theoretical framework requires testing by empirical research.

**Summary:**

Implementation fidelity is an important source of variation affecting the credibility and utility of research. The conceptual framework presented here offers a means for measuring this variable and understanding its place in the process of intervention implementation.

## Background

Implementation fidelity is "the degree to which . . . programs are implemented . . . as intended by the program developers" [1]. This idea is sometimes also termed "integrity" [1,2]. Implementation fidelity acts as a potential moderator of the relationship between interventions and their intended outcomes. That is to say, it is a factor that may impact on the relationship between these two variables (*i.e*., how far an intervention actually affects outcomes. This is one of the principal reasons why implementation fidelity needs to be measured. It has been demonstrated that the fidelity with which an intervention is implemented affects how well it succeeds [1-5]. For instance, two studies examining programmes to help people with mental health issues obtain employment found that employment outcomes among their study groups were weakest for those in poorly implemented programmes [6,7]. In the same way, a study of a parent training programme found that when the programme was implemented with high fidelity, the parenting practices improved significantly, but the effect was much less when implementation fidelity was low [8]. Other recent studies have found similar associations [9,10].

It is only by making an appropriate evaluation of the fidelity with which an intervention has been implemented that a viable assessment can be made of its contribution to outcomes, *i.e*., its effect on performance. Unless such an evaluation is made, it cannot be determined whether a lack of impact is due to poor implementation or inadequacies inherent in the programme itself, a so-called Type III error [11]; this is also addressed by the thesis of comprehensiveness [12]. It would also be unclear whether any positive outcomes produced by an intervention might be improved still further, if it were found that it had not been implemented fully.

Primary research into interventions and their outcomes should therefore involve an evaluation of implementation fidelity if the true effect of the intervention is to be discerned. Moreover, evidence-based practitioners also need to be able to understand and quantify the fidelity with which they are implementing an intervention. Evidence-based practice assumes that an intervention is being implemented in full accordance with its published details. This is particularly important given the greater potential for inconsistencies in implementation of an intervention in real world rather than experimental conditions. Evidence-based practice therefore not only needs information from primary researchers about how to implement the intervention, if replication of the intervention is to be at all possible, it also needs a means of evaluating whether the programme is actually being implemented as the designers intended.

Similar issues affect secondary research: the common lack of data on implementation fidelity provided by primary research studies, known as "thinness", prevents those working on systematic reviews and meta-analyses from gauging possible heterogeneity between studies, with the result that data may be pooled or aggregated inappropriately [13,14]. Consequently, the internal validity of a review may be adversely affected, and, thus, the credibility and utility of that research may be thrown into question. The degree of implementation fidelity achieved by an intervention may also explain why some studies generate different results, even though they appear to be the same in every other way.

In summary, evaluation of implementation fidelity is important because this variable may not only moderate the relationship between an intervention and its outcomes, but its assessment may also prevent potentially false conclusions from being drawn about an intervention's effectiveness. It may even help in the achievement of improved outcomes. It can give primary researchers confidence in attributing outcomes to the intervention; evidence-based practitioners can be confident they are implementing the chosen intervention properly; and secondary researchers can be more confident when synthesising studies. This all requires a framework within which to understand and measure the concept and process of implementation fidelity. Accordingly, the objective of this paper is to critically review the literature on implementation fidelity, particularly within primary research – the implementation of an intervention – and to propose a new framework for understanding and evaluating this concept.

### Conceptualisations of implementation fidelity and their limitations

A search was performed to identify literature on implementation fidelity, *i.e*., empirical research, reviews, or theoretical pieces. The following databases were searched with the terms "implementation fidelity" or "fidelity" within five words of "implement", "implementation", "implemented", etc.: Applied Social Sciences Index and Abstracts, Cumulative Index of Nursing and Allied Health Literature (CINAHL), International Bibliography of the Social Sciences, MEDLINE, and the Social Science Citation Index. The relevant studies identified by this search were also then scanned for additional literature. Conference abstracts and presentations provided another source of literature in this field. This multi-method search identified a number of reviews discussing the conceptualisation of implementation fidelity, and a body of empirical research measuring the fidelity with which various interventions had been implemented. This article focuses principally on research published from 2002 to 2007, because of the presence of major reviews of the implementation fidelity literature from 2003 and before [1,2,4,5]. The arguments, limitations, and findings of all of this literature contributed to the development of the novel framework presented here and provided examples of how to evaluate individual elements of the framework.

### A conceptual framework: Background and Rationale

The concept of implementation fidelity is currently described and defined in the literature in terms of five elements that need to be measured [1,2,4]. These are: adherence to an intervention; exposure or dose; quality of delivery; participant responsiveness; and programme differentiation. There are certain overlaps here with the concept of process evaluation [15]. Within this conceptualisation of implementation fidelity, adherence is defined as whether "a program service or intervention is being delivered as it was designed or written" [4]. Dosage or exposure refers to the amount of an intervention received by participants; in other words, whether the frequency and duration of the intervention is as full as prescribed by its designers [1,4]. For example, it may be that not all elements of the intervention are delivered, or are delivered less often than required. Coverage may also be included under this element, *i.e*., whether all the people who should be participating in or receiving the benefits of an intervention actually do so.

Quality of delivery is defined as "the manner in which a teacher, volunteer, or staff member delivers a program" [4]. However, it is perhaps a more ambiguous element than this suggests. An evaluation of this may require using a benchmark, either within or beyond that stipulated by an intervention's designers; this element of fidelity could involve either delivering the intervention using "techniques . . . prescribed by the program" [4], or applying a benchmark from outside the programme, *i.e*., "the extent to which a provider approaches a theoretical ideal in terms of delivering program content" [1]. If such a clear benchmark exists then quality of delivery may be treated, along with adherence and dosage, as one of three discrete aspects required to assess the fidelity of an intervention. However, it may potentially also be viewed as a moderator of the relationship between an intervention and the fidelity with which it is implemented. This is a role that is simply not explored in the literature to date. For example, an intervention could be delivered but delivered badly; in turn, the degree of fidelity achieved by the implemented intervention could be adversely affected.

Participant responsiveness measures how far participants respond to, or are engaged by, an intervention. It involves judgments by participants or recipients about the outcomes and relevance of an intervention. In this sense, what is termed "reaction evaluation" in the evaluation literature may be considered an important part of any evaluation of an intervention [16].

Program differentiation, the fifth aspect, is defined as "identifying unique features of different components or programs", and identifying "which elements of . . . programmes are essential", without which the programme will not have its intended effect [1]. Despite being viewed as an element of implementation fidelity by the literature, programme differentiation actually measures something distinct from fidelity. It is concerned with determining those elements that are essential for its success. This exercise is an important part of any evaluation of new interventions. It enables discovery of those elements that make a difference to outcomes and whether some elements are redundant. Such so-called "essential" elements may be discovered either by canvassing the designers of the intervention or, preferably, by "component analysis", assessing the effect of the intervention on outcomes and determining which components have the most impact [17]. This element would therefore be more usefully described as the "Identification of an intervention's essential components". This process may also have implications for implementation fidelity; if, for example, these essential components are the most difficult to implement, then this may then explain a lack of success afflicting the intervention.

Despite agreeing that implementation fidelity involves measurement of these five elements, the review literature offers two distinct views on how this should be done. On the one hand, it is argued that each of these five elements represents an alternative way to measure fidelity, *i.e*., implementation fidelity can be measured using either adherence or dosage or quality of delivery etc [4,5]. On the other hand, it is argued that all five elements need to be measured to capture a "comprehensive" or "more complete picture" of the process, *i.e*., evaluation requires the measurement of adherence, dosage, and quality of delivery, etc [1,2]. However, relationships between the various elements are far more complex than such conceptualisations allow. This paper therefore advances a new, third conceptual framework for implementation fidelity, which not only proposes the measurement of all of these elements, but unlike all previous attempts to make sense of this concept also clarifies and explains the function of each and their relationship to one another. Two additional elements are also introduced into this new framework: intervention complexity and facilitation strategies. The potential effect of intervention complexity on implementation fidelity was suggested to the authors by literature on implementation more broadly – especially a systematic review that focused on identifying facilitators and barriers to the diffusion of innovations in organisations that found that the complexity of an idea presented a substantial barrier to its adoption [18]. The potential role of facilitation strategies was suggested by research aiming to evaluate the implementation fidelity of specific interventions that put in place strategies to optimise the level of fidelity achieved. Such strategies included the provision of manuals, guidelines, training, monitoring and feedback, capacity building, and incentives [3,6,8,17].

### Proposed framework

**Figure 1:**
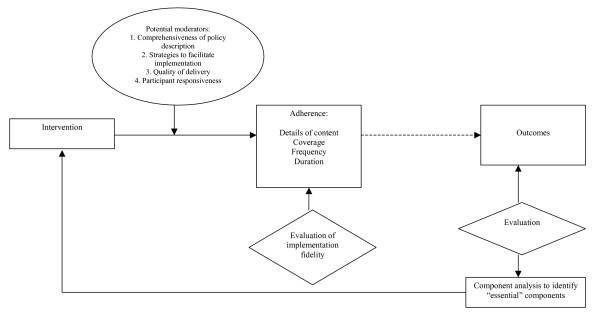
Conceptual framework for implementation fidelity.

All of the elements to evaluate implementation fidelity are listed in Table 1, and the relationships between them are shown in the framework depicted in Figure 1.

**Table 1:** 

**Elements of implementation fidelity**
Adherence
- Content
- Coverage
- Frequency
- Duration
-

Moderators
- Intervention complexity
- Facilitation strategies
- Quality of delivery
- Participant responsiveness

Identification of essential components

The framework outlined in Figure 1 depicts the vital elements of implementation fidelity and their relationship to one another. The measurement of implementation fidelity is the measurement of adherence, *i.e*., how far those responsible for delivering an intervention actually adhere to the intervention as it is outlined by its designers. Adherence includes the subcategories of content, frequency, duration and coverage (*i.e*., dose). The degree to which the intended content or frequency of an intervention is implemented is the degree of implementation fidelity achieved for that intervention. The level achieved may be influenced or affected, (*i.e*., moderated) by certain other variables: intervention complexity, facilitation strategies, quality of delivery, and participant responsiveness. For example, the less enthusiastic participants are about an intervention, the less likely the intervention is to be implemented properly and fully.

The broken line in Figure 1 indicates that the relationship between an intervention and its outcomes is external to implementation fidelity, but that the degree of implementation fidelity achieved can affect this relationship. Finally, an analysis of outcomes may identify those components that are essential to the intervention, and must be implemented if the intervention is to have its intended effects. This evaluation in turn may inform the content of the intervention by determining the minimum requirements for high implementation fidelity, *i.e*., the implementation of the essential components of the intervention. The following discussion describes the function of each element in detail, highlighted by examples from the research. Relationships between moderators are also considered.

### A conceptual framework: elements and relationships

#### Adherence

Adherence is essentially the bottom-line measurement of implementation fidelity. If an implemented intervention adheres completely to the content, frequency, duration, and coverage prescribed by its designers, then fidelity can be said to be high. Measuring implementation fidelity means evaluating whether the result of the implementation process is an effective realisation of the intervention as planned by its designers.

The content of the intervention may be seen as its 'active ingredients'; the drug, treatment, skills, or knowledge that the intervention seeks to deliver to its recipients. For example, the Marriage and Parenting in Stepfamilies parent training programme is based on thirteen sessions, each with specific materials to be delivered to parents by trained educators [8]. The programme has a number of designated components, such as skill encouragement and discipline. The fidelity with which this intervention was implemented, *i.e*., the level of adherence to its model, was evaluated by trained coders using videotapes of sessions to score how far the implemented intervention actually adhered to the prescribed model in terms of content [8]. The tool used in this study was the Fidelity of Implementation Rating System (FIMP). Observation by trained staff of those delivering the intervention is often used to evaluate fidelity [19,20].

Subcategories of adherence concern the frequency, duration, or coverage of the intervention being delivered, *i.e*., what is more broadly defined as "dose" in the existing literature. For example, one violence prevention programme used interviews and surveys with staff to determine whether the intervention had been implemented as often and for as long as prescribed, and found that high implementation fidelity was only achieved in a little over one-half of cases [3]. In the same way, an evaluation of the implementation of a residential treatment programme for adolescents with substance abuse issues required staff to keep a log of the number of hours of services provided by the Adolescent Treatment Program, and this was compared with the number of hours prescribed by the intervention's model [21]. Implementation fidelity was found to be relatively low, with only about one-half of the required time being spent in the activities prescribed by the intervention.

The measurement of adherence to an intervention's predefined components can therefore be quantifiable: An evaluation to gauge how much of the intervention's prescribed content has been delivered, how frequently, and for how long. However, adherence may not require every single component of an intervention to be implemented. An intervention may also be implemented successfully, and meaningfully, if only the "essential" components of the model are implemented. However, the question remains about how to identify what is essential. One possible way to do this is to conduct a sensitivity analysis, or "component analysis", using implementation fidelity data and performance outcomes from different studies of the same intervention to determine which, if any, components or combination of components are essential, *i.e*., are prerequisite if the intervention is to have its desired effect. However, if essential components of an intervention are not known, then fidelity to the whole intervention is needed.

Identifying these essential components also provides scope for identifying adaptability to local conditions. An intervention cannot always be implemented fully in the real world. Local conditions may require it to be flexible and adaptable. Some specifications of interventions allow for local adaptation. Even if they do not explicitly do this, local adaptations may be made to improve the fit of the intervention within the local context. Indeed, the pro-adaptation perspective implies that successful interventions are those that adapt to local needs [22]. However, some argue that the case for local adaptation may well have been exaggerated, at least for interventions where the evidence does not necessarily support it [3]. The intermediate position is therefore that programme implementation can be flexible as long as there is fidelity to the so-called "essential" elements of an intervention. The absence of these elements would have significant adverse effects on the capacity of an intervention to achieve its goals. Indeed, without them it cannot meaningfully be said that an intervention has achieved high implementation fidelity.

#### Moderators

A high level of adherence or fidelity to an intervention, or its essential components, is not achieved easily. Several factors may influence or moderate the degree of fidelity with which an intervention is implemented. Each of the potential moderators of this relationship is now considered in turn.

#### Intervention complexity

The description of an intervention may be simple or complex, detailed or vague. Detailed or specific interventions have been found to be more likely to be implemented with high fidelity than ones that are vague. For example, a study of guidelines intended for General Practitioners (GPs) found that detailed and clear recommendations were almost twice as likely to be followed as vague and non-specific recommendations [23]. The specificity of these guidelines was assessed by a group of researchers and their uptake was evaluated by the GPs' self-report. In the same way, well-planned interventions, where all the key components are identified in advance, have been found to produce higher levels of adherence than less well-structured interventions [1,5]. Specificity enhances adherence.

There is also evidence that it is easier to achieve high fidelity of simple than complex interventions [1]. This may be because there are fewer "response barriers" when the model is simple [18]. Complex interventions have greater scope for variation in their delivery, and so are more vulnerable to one or more components not being implemented as they should. This has led to calls in some quarters for improving the recording and reporting of complex interventions to identify and address potential sources of heterogeneity in implementation [13,14,24]. Overall, research suggests that simple but specific interventions are more likely to be implemented with high fidelity than overly complex or vague ones. As such, the comprehensiveness and nature of an intervention's description may influence how far the programme successfully adheres to its prescribed details when implemented.

#### Facilitation strategies

Support strategies may be used both to optimise and to standardise implementation fidelity, *i.e*., to ensure that everyone is receiving the same training and support, with the aim that the delivery of the intervention is as uniform as possible [25]. Such strategies include the provision of manuals, guidelines, training, and monitoring and feedback for those delivering the intervention.

Some studies that evaluate the implementation process have monitored the extent to which an intervention is being implemented correctly, and then have fed back these results to those delivering the intervention. A study measuring fidelity to an exercise programme for women with hip fractures used direct observation by the designers of the intervention to monitor the intervention that was actually being delivered, and then provided feedback to the exercise trainers [21]. In this way, deviations from the intended content of the programme were addressed and corrected, and high fidelity was achieved.

It is therefore possible that these strategies, like the nature of an intervention's description, may potentially moderate the degree of fidelity achieved: the more that is done to help implementation, through monitoring, feedback, and training, the higher the potential level of implementation fidelity achieved. The role of such strategies in optimising fidelity and standardising what is being implemented is arguably even more important in the case of complex interventions, which can be multifaceted and therefore more vulnerable to variation in their implementation [24]. Although some studies have claimed that the provision of certain facilitation strategies has positively affected implementation of an intervention, these claims are not the result of empirical research [13]. However, no study has yet measured the moderating effect of these strategies on the degree of implementation fidelity.

More facilitation strategies do not necessarily mean better implementation. A simple intervention may require very little in terms of training or guidance to achieve high implementation fidelity. A complex intervention by contrast may require extensive support strategies. There is therefore an issue of adequacy, and this may be determined by the relationship between facilitation strategies and the complexity of an intervention's description. The relationship between these potential moderators is discussed more fully below. Empirical research has yet to demonstrate whether facilitation strategies can indeed affect how well or how badly an intervention is implemented, but this should certainly be considered as a potential moderator of implementation fidelity.

#### Quality of delivery

Quality of delivery is an obvious potential moderator of the relationship between an intervention and the fidelity with which it is implemented. It concerns whether an intervention is delivered in a way appropriate to achieving what was intended. If the content of an intervention is delivered badly, then this may affect the degree to which full implementation is realised. In studies evaluating fidelity the provision of extensive training, materials and support to those delivering an intervention is an implicit acknowledgement that effort is required to optimise the quality of the delivery of the intervention being evaluated [3,26-28]. In the same way, quality assurance or improvement strategies, such as providing ongoing monitoring and feedback to those delivering the intervention, provide a more explicit acknowledgement of the importance of quality of delivery and its potential moderating effect on implementation fidelity [28,29].

A study of the implementation of a parent training programme included quality of teaching in its Fidelity of Implementation Rating System (FIMP) [8]. This involved assessments by trained observers to determine whether the parent trainers applied both verbal and active teaching strategies, as required by the intervention. The scale stipulated that an "Over-reliance on verbal teaching can result in lower scores". Trained observers were also used to assess both content and process fidelity, including quality of delivery, of a life skills training program delivered by teachers in the United States [19]. However, these studies did not analyse quality of delivery as a moderator of implementation fidelity, but rather as a discrete aspect of fidelity.

#### Participant responsiveness

If participants view an intervention as being of no relevance to them, then their non-engagement may be a major cause of its failure or low coverage, and thus implementation fidelity may be low. This idea – that the uptake of a new intervention depends on its acceptance by and acceptability to those receiving it – echoes Rogers' diffusion of innovations theory [30]. Participant responsiveness may therefore be an important moderator in any process examining implementation fidelity. For example, it has been found that implementation fidelity of prescribed drug interventions for elderly people in the community can be low because these patients deliberately fail to comply with their prescribed regimens [31-33]. Reasons for this intentional non-compliance include the unpleasant side effects of the drugs, and because the therapy is only preventative or symptoms only mild, so patients feel less inclined to comply [31-33]. In a study of a school-based health promotion intervention, the teachers reported that they did not implement certain components of the intervention if they felt the students were not responding and were not interested [34].

In fact, participants covered by this moderator of implementation fidelity encompass not only the individuals receiving the intervention, but also those responsible for it. For example, studies examining factors associated with substance abuse prevention and health promotion programmes in schools found that teachers' beliefs concerning the intervention itself, for example whether they liked it or not, and the training and support they themselves had received, were all associated with their level of adherence to the intervention [34,35]. In other words, higher levels of implementation fidelity were achieved when those responsible for delivering an intervention were enthusiastic about it. The organisation more broadly may also influence the response of those delivering a new intervention. If an organisation, as represented by senior management for example, is not committed to an intervention, then the responsiveness of individuals may be affected, too [2]. This is a key aspect of all organisational change literature [36].

Self-report is the most common means of evaluating the responsiveness of all participants to an intervention [30-34,37]. This assessment can involve several perspectives. It may evaluate how far participants fully accept the responsibilities required by an intervention [38], how far they perceive the intervention to be useful [26] and, more broadly, how responsive the environment is into which an intervention is introduced, the so-called "therapeutic milieu", which may not be conducive to a favourable response from participants [21]. In studies that have examined these dimensions of participant responsiveness, participants used logs and calendars to record and report on their response to the intervention being implemented. Participant responsiveness may even reach beyond attitudes to actual action, for example, to gauge whether a "treatment has been . . . understood . . . and that the individual performs treatment related . . . . skills and strategies" [29]. In this sense, "enactment" may be considered a potential element of participant responsiveness [25].

#### Relationships between moderators

These moderators are not necessarily discrete elements. There may be relationships at work between two or more moderators. An obvious example is where the provision of training and guidelines on how to deliver an intervention may have a direct impact on the quality with which an intervention is actually delivered (and this may in turn affect the fidelity with which an intervention is implemented). If the amount of training provided is small, then the quality of the resulting delivery may be poor. Facilitation strategies may also influence participant responsiveness: The provision of incentives could make both providers and participants more amenable or responsive to a new intervention. Quality of delivery may function in the same way: a well-delivered intervention may make participants more enthusiastic and committed to it. One moderator might therefore predict another.

However, as noted above, these relationships are more complex than may be captured in the simple correlation of large numbers of facilitation strategies producing high quality of delivery, or by stating that small incentives produce limited participant responsiveness. One reason is the moderating role of intervention complexity: A simple intervention may not require much training or guidance to achieve high quality of delivery or participant responsiveness. A small amount of training may suffice. In other words, there may be interaction effects between moderators, *i.e*., when the effect of one factor is dependent on the level of another. Participants may also be enthusiastic about new interventions because of other factors, regardless of incentives or other strategies.

Thus the interaction of these moderators may further affect the relationship between an intervention and the fidelity with which it is implemented.

#### Measurement

The implication of our framework is that any evaluation must measure all the factors listed above that influence the degree of implementation fidelity, such as intervention complexity and the adequacy of facilitation strategies. It also needs to gauge participant responsiveness or receptiveness to proposed and implemented interventions. With the exception of a few studies that do measure quality of delivery or participant responsiveness [8,20,38], most implementation fidelity research focuses solely on a fidelity score determined almost exclusively by adherence [3,6-8,21,22,27-29,38,39]. Moreover, this research rarely reports high implementation fidelity [8,29,40]. It actually often falls short of the ideal and is sometimes even very poor, yet it is only by measuring the moderators described above that potential explanations for low or inadequate implementation may be apprehended or understood. It is only by identifying and controlling for the contribution of possible barriers to implementation that such issues can be addressed and higher implementation achieved.

## Summary

Achievement of high implementation fidelity is one of the best ways of replicating the success with interventions achieved by original research. Successful evidence-based practice is governed by many things [41], and implementation fidelity is one of them. This paper offers a more complete conceptual framework for implementation fidelity than proposed hitherto, and explains why and how implementation fidelity should be evaluated. The framework is multifaceted, encompassing both the intervention and its delivery. Adherence relates to the content and dose of the intervention, *i.e*., has the content of the intervention – its 'active ingredients' – been received by the participants as often and for as long as it should have been. However, the degree to which full adherence, *i.e*., high implementation fidelity, is achieved may be moderated by factors affecting the delivery process, such as facilitation strategies, quality of delivery, and participant responsiveness.

This conceptualisation provides researchers with a potential framework for implementation research. Monitoring of implementation fidelity following this framework enables better evaluation of the actual impact of an intervention on outcomes. In turn, the credibility and utility of the resulting research would be enhanced accordingly. It also offers evidence-based practitioners a guide to the processes and factors at play when implementing interventions described in research. However, much more research is needed on this topic. Empirical research is needed to test the framework itself and to clarify the moderating impact of the components included here.

## Competing interests

The author(s) declare that they have no competing interests.

## Authors' contributions

CC drafted the paper; CC, MP, and SW are responsible for the intellectual content of the paper. All authors approved the final manuscript.
